# Mental Health Care Guidelines for Telemedicine During the COVID-19 Pandemic: Scoping Review

**DOI:** 10.2196/56534

**Published:** 2025-07-09

**Authors:** Julia Ivanova, Triton Ong, Hattie Wilczewski, Mollie Cummins, Hiral Soni, Janelle Barrera, Brandon Welch, Brian Bunnell

**Affiliations:** 1 Doxy.me Research Doxy.me, Inc Charleston, SC United States; 2 College of Nursing Department of Biomedical Informatics University of Utah Salt Lake City, UT United States; 3 Department of Psychiatry and Behavioral Neurosciences University of South Florida Tampa, FL United States; 4 Biomedical Informatics Center Public Health and Sciences Medical University of South Carolina Charleston, SC United States

**Keywords:** telemedicine, telehealth, guideline, mental health, health care, COVID-19, pandemic, scoping review, thematic analysis, mental health, telemental care, screening, effectiveness, eHealth, digital health, online care, online health

## Abstract

**Background:**

Mental health care providers have widely adopted telemedicine since the onset of the COVID-19 pandemic. Some providers have reported difficulties in implementing telemedicine and are still assessing its sustainability for their practices. Recommendations, best practices, and guidelines for telemedicine-based mental health care (ie, telemental health care [TMH]) have been published, but the nature and extent of this guidance have not been assessed.

**Objective:**

We aimed to determine (1) the form of TMH guidelines and recommendations presented to providers, (2) the most commonly presented recommendations and guidelines, and (3) the perceived benefits and challenges of these TMH guidelines and recommendations.

**Methods:**

Through our scoping review of practice guidelines, we aimed to identify themes in TMH guidelines and clinical recommendations published between 2020 and 2024 in peer-reviewed journals. This review focused on the first 2 years of the COVID-19 pandemic to identify and characterize the available TMH guidance. We searched PubMed/MEDLINE and ScienceDirect for articles in peer-reviewed journals published between January 1, 2020, and July 16, 2024. We included articles that were available in English and presented recommendations, best practices, or guidelines for TMH. We excluded duplicates, articles unrelated to telehealth, brief editorial introductions, and those not publicly available. We applied the Healthcare Provider Taxonomy of the National Uniform Claim Committee to article titles and abstracts to identify records relevant to mental health. We used content and thematic analyses to identify key themes.

**Results:**

Of the 1348 articles retrieved, we identified 76 that matched our criteria. Through content and thematic analyses, we identified 3 main themes—along with subthemes and topics—related to Facilitators, Concerns, and Changes Advised. The majority of articles called for further research (59/76) and for telemental health education and innovation in some form (43/76) regarding advised changes. Twenty-four articles included specific guidelines, recommendations, or checklists for providers.

**Conclusions:**

The results highlight the need for further large-scale research to support the development of effective guidelines and protocols for therapy plans. Although TMH care is widespread, scholarly work emphasizes the need for a stronger evidence base that includes testing protocols in diverse settings and populations. The results also underscore the importance of increasing health professionals’ knowledge of regulatory compliance and providing them with adequate TMH practice education.

## Introduction

Telemedicine was quickly and widely adopted in early 2020 to curb the spread of the SARS-CoV-2 virus and to increase access to health care [1]. As health care professionals used telemedicine to limit the spread of COVID-19 in hospitals, clinics, and private practices, its role extended beyond outbreak control and became a widely accepted means of delivering care [2]. With the boom in telemedicine during the COVID-19 pandemic [1], the need for support, guidelines, and recommendations quickly became apparent, as new users struggled to use telemedicine effectively [3]. Mental health providers have shown strong support for telemedicine and remain its most active users, even beyond the initial impact of the pandemic [4-6]. While many providers discontinued telemedicine for physical health conditions after June 2020, mental health providers have continued to use it well beyond the early months of COVID-19 [7]. Due to the nature of their practice—based primarily on interpersonal communication—mental health care providers adopted telemedicine more readily than providers in specialties that rely more heavily on interventions and physical examinations, such as dermatology, cardiology, or primary care [4, 8, 9]. A review of telemedicine guidelines and recommended best practices for mental health providers—herein referred to as telemental health care (TMH) guidelines—may offer insight into how providers can best utilize telemedicine and what changes to those guidelines may still be necessary.

With the introduction of the Coronavirus Aid, Relief, and Economic Security Act and Public Health Emergency protocols in the United States, telemedicine was irrevocably transformed by newfound flexibilities and waivers [10]. Similar regulatory loosening occurred worldwide, as governing bodies supported the medical community’s efforts to manage the pandemic through telemedicine [11]. While this flexibility made telemedicine more accessible to both providers and patients, it also rendered some previously established telemedicine guidelines and internal policies obsolete, creating the need for new, updated guidance [11]. In a 2022 systematic review of telemedicine challenges faced by health care professionals during the pandemic, technological prerequisites were identified as the most common barrier to providing care via telemedicine, followed by concerns such as security and privacy, training and education for both providers and patients, and the provider-patient relationship [12]. Naturally, clinical guidelines tend to be specific to a specialty or disease; this specificity means, for example, that mental health professionals use telemedicine guidelines tailored expressly to their field.

Medical guidelines aim to establish steps and criteria that make care delivery systematic and ensure the highest quality of care for patients [13, 14]. Ideally, TMH providers would have access to such guidelines, covering expectations for care delivery, legal compliance, and technical considerations [15]. TMH guidelines—including those from the American Psychological Association (APA) and the American Telemedicine Association (ATA)—already existed within the mental health community before the pandemic [16, 17]. Other guidelines, such as the guide from the Substance Abuse and Mental Health Services Administration (SAMHSA) in the United States, were published during the COVID-19 pandemic and were not initially available to providers [18]. While TMH guidelines did exist before the pandemic and were updated between 2020 and 2024, a review of what providers were able to access in peer-reviewed sources may shed light on TMH providers’ needs—particularly those based on original research and other forms of evidence more readily captured in scholarly publications.

In this scoping review of the relevant biomedical literature, we aimed to answer the following questions: (1) What forms of guidelines and recommendations were presented to providers (eg, checklists, step-by-step user guides, lists of best practices)? (2) What recommendations and guidelines are most commonly presented in academic literature for providers to be aware of in TMH practice? and (3) What are the perceived benefits and challenges of TMH guidelines and recommendations?

## Methods

### Search Strategy

We conducted a scoping review of the biomedical literature, following the Journal of Biomedical Informatics’ updated methodological guidance [2] and procedures from similar scoping reviews, including formal content and thematic analyses [19-21]. As we were interested in identifying and understanding the nature of published TMH guidelines during the pandemic, we conducted a scoping review with qualitative analysis to identify and synthesize the emerging literature. As a preliminary step, we reviewed existing literature to ensure that our research aims and timeline had not already been addressed. We searched the PubMed/MEDLINE and ScienceDirect databases for reviews using keywords related to our aims and found none [22].

We searched the PubMed/MEDLINE and ScienceDirect databases on March 14, 2022, and again on July 16, 2024. Search strings included “(tele*[Title]) AND (guid*[Title])” and “(tele*[Title]) AND (practic*[Title])” in Pubmed/MEDLINE, “(telemedicine OR telehealth) AND (guide OR guidance OR guiding)” in ScienceDirect, and “(telemedicine OR telehealth) AND (practice OR practical OR practicing)” in ScienceDirect. We limited search results to articles published between January 1, 2020, and July 16, 2024, and restricted the search to titles only. No other restrictions were applied. Three researchers (TO, JI, and HW) conducted the searches and used database features to export the results into CSV files, which were then merged into an Excel spreadsheet (Microsoft Corporation). The search results from the 3 researchers were compared to ensure consistency.

### Article Selection

We included articles if they were available in English and provided recommendations, best practices, or guidelines for mental health via telemedicine. We excluded articles if they were duplicates, if their primary focus was unrelated to TMH care, if the article was a brief editorial introduction to a particular journal section, or if the entire article could not be located through the University of Florida library or interlibrary loan service. We applied the Healthcare Provider Taxonomy of the National Uniform Claim Committee [23] to article titles and abstracts to identify records relevant to mental health. This was done to focus solely on work directly defined as mental health. One researcher (TO) applied the group/individual taxonomy to each article title; in cases where the title was ambiguous, the researcher also considered the abstract. The research team reviewed the results for accuracy. Two researchers (TO and JI) independently reviewed the articles for selection. Final exclusions were made during the full-text review.

### Data Extraction and Analysis

We extracted the entire content of the articles, including guidelines and recommendations for TMH health care practice, for qualitative analysis. We then conducted both content analysis and thematic analysis [3-5]. The content of each article was reviewed to identify principles, proposals, suggestions, advice, or considerations to promote TMH practice, using MAXQDA (VERBI GmbH), a qualitative coding and analytic platform [6]. Two researchers (TO and JI) independently reviewed the articles to identify practice guidelines and key themes through content and thematic analyses [21, 24-28].

One researcher (JI) coded each full-text article based on the 3 questions above. The content analysis focused on (1) changes advised or guidelines recommended for TMH providers (research questions 1 and 2), (2) facilitators and benefits related to telemedicine usage (research question 3), and (3) concerns and barriers to telemedicine usage (research question 3) [26]. These 3 categories were chosen because they generally capture topics found in the guidelines and recommendations of the resulting articles. We developed a codebook based on the expected definitions of these topics and revised the codebook with each iteration of coding (see Table 1 for the codebook).

After the initial content analysis, a single reviewer (JI) completed another iteration of coding using thematic analysis to capture emergent topics within the 3 content analysis codes. The thematic analysis focused on items already identified in the content analysis, using techniques such as repetition of concepts, typology, metacoding, missing data, and cutting and sorting [26, 28]. For all coding, the unit of analysis was meaningful phrases—including bulleted lists and entire paragraphs. One author (JI) completed 3 iterations of content and thematic analyses. The codebook and coding were reviewed and refined by the research team for consistency and accuracy (see Table 1 for the codebook with exemplar quotes). Using frequency by recording and metacoding, we categorized the codes into themes, subthemes, and topics [27].

Given the generalized focus on characterizing the existing literature on TMH guidelines and recommendations, the scoping review does not include a formal quantitative synthesis or an assessment of the risk of bias. Instead, we used the results from content and thematic analyses—in the form of themes, subthemes, and topics—to answer our research questions.

## Results

### Overview of Identified and Included Literature

**Figure 1:**
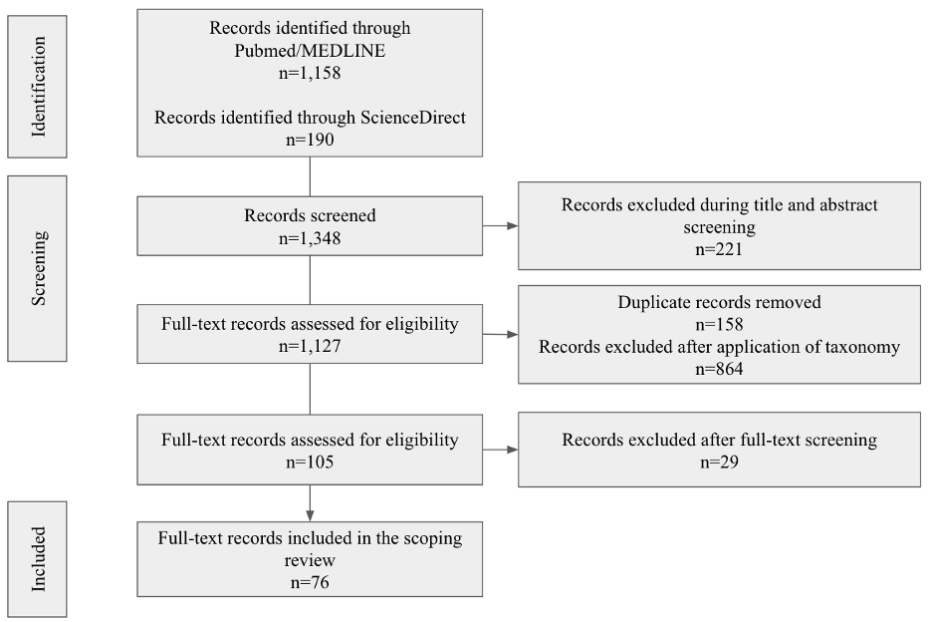
PRISMA (Preferred Reporting Items for Systematic Reviews and Meta-Analyses) schema showing results of the literature search and resulting exclusions and inclusions.

Our initial search identified more than 1300 articles (see Figure 1 and Multimedia Appendix 1). We excluded 158 duplicates and 221 articles during the title and abstract screening, resulting in 969 articles for full-text review. By applying the Healthcare Provider Taxonomy of the National Uniform Claim Committee, we excluded 864 articles related to other medical specialties, focusing on 105 articles for TMH guidelines [23]. During the full-text review, we excluded an additional 29 articles: 8 due to the inability to access the full text and 21 because the article topic did not meet the inclusion criteria. We exported the final 76 articles to MAXQDA for content and thematic analyses.

The final articles were predominantly written in 2020 (28/76, 37%) and 2021 (28/76, 37%), with 11 published in 2022, 8 in 2023, and 1 in 2024. We found only a handful of journals that repeatedly published articles relevant to the scoping review: Australasian Psychiatry (n=5), Archives of Clinical Neuropsychology (n=5), Journal of the American Psychiatric Nurses Association (n=4), Indian Journal of Psychological Medicine (n=5), Behavior Analysis in Practice (n=3), Telemedicine and e-Health (n=2), Cognitive and Behavioral Practice (n=2), JMIR Mental Health (n=2), Contemporary Family Therapy (n=2), International Journal of Medical Informatics (n=2), and Indian Journal of Psychiatry (n=2). Over half of the resulting articles were original research (39/76, 51%), followed by viewpoints and reviews (15/76, 20%), guides and suggestions (13/76, 17%), and systematic or scoping reviews (9/76, 12%). A total of 27 original research articles were published between 2020 and 2021, and 12 were published between 2022 and 2024. We found 47 different organizations cited by the resulting articles, with the APA referenced 10 times [7-13, 16-18], the ATA mentioned 3 times [8, 9, 17], and the International Organization of Psychophysiology and Cognitive Sciences (IOPC) [13, 17], Centers for Medicare & Medicaid Services (CMS) [9, 12], Centers for Disease Control and Prevention (CDC) [9, 12], Institute of Medicine [14, 16], and Telemedicine and Psychiatry Operational Guidelines 2020 cited 2 times [15, 19].

### Overview of Qualitative Analytic Results

**Figure 2:**
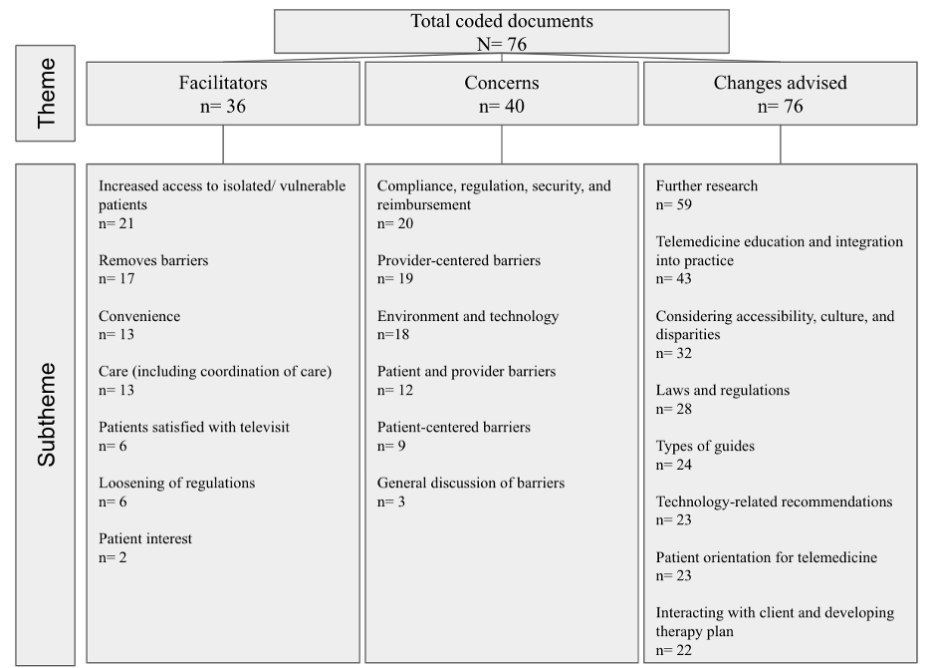
Themes by total articles and frequency. Subthemes by total total articles.

Content and thematic analyses yielded 2483 codes, with the majority found in Changes Advised (1366/2483, 55.01%). For this scoping review, however, we focus on code presence within a record rather than total frequency (see Figure 2). While all resulting records had Changes Advised codes, 40 out of 76 (53%) included specific Concerns, and 36 out of 76 (47%) included Facilitators (see Figure 2 and Table 1 for the codebook). Over the 3 iterations of coding, we identified 8 subthemes in Changes Advised, 7 in Facilitators, and 6 in Concerns. Each subtheme included topics of discussion relevant to the subtheme.

**Table 1:** Codebook with exemplar quotes from cited articles.

Themes and subthemes			n		Citations		Definition		Example quote
**Facilitators**			36				Mention of TMH’s^a^ positive aspects that can be leveraged for health care delivery		“A commonly reported benefit of telemedicine included increasing patient access to care and its most important feature was HIPAA compliance, followed by being low-cost and easy-to-use [20].”
	Increased access to isolated/vulnerable patients	21		[8-10, 14, 16, 17, 19-22, 24-34]		The role of TMH in accessibility to care for patients, especially those who are socioeconomically vulnerable		“...it is not yet clear whether telehealth opportunities have done much to expand treatment accessibility beyond populations who already had no difficulty reaching mental health care prior to the pandemic. One of the great promises of telehealth is its potential to overcome barriers and reach new, previously under-served populations [28].”	
	Removes barriers (cost, transport, time, and stigma)	17		[8, 9, 14, 16, 21, 22, 25, 27, 30-38]		The role of TMH in easing known barriers to in-person visits such as decreasing cost, time, and stigma and easing worries over transportation and child care		“These services are cost-effective due to increased productivity, time management, and reduces transportation expenses [16].”	
	Convenience of telemental health care	13		[8, 9, 16, 17, 21, 22, 30, 32-35, 37, 39]		Discussion of TMH in terms of “convenience” or ease of use for providers		“There are indications that the satisfaction by providers is even higher today now that there is the opportunity to connect from the convenience of their home (Kocsis & Yellowlees, 2018; Yellowlees, 2020) [9].”	
	Care (including coordination of care)	13		[8, 9, 11, 16, 21, 22, 26, 28, 32, 33, 37, 40, 41]		Discussion of TMH in regard to coordination and continuity of care for patients		“The APA Guidelines and the AAP Procedures discuss the necessity of assessing the continuity and termination of care on a regular basis in order to maintain quality service via telehealth. It is noted that ABA practitioners are in a unique position compared to these professionals in that the frequency and the duration of telehealth sessions are higher [11].”	
	Loosening of regulations	6		[24, 28, 39, 42-44]		Mention of telemedicine regulatory flexibilities due to the pandemic as a facilitator for its use		“In the United States, a number of federal and state emergency orders enacted in the early weeks of the pandemic, and a number of expanded reimbursement policies, cleared the way for telehealth to take over as the primary strategy for maintaining continuity of care across the pandemic [28].”	
	Patients satisfied with telemental visit	6		[8, 16, 24, 31, 34, 37]		Patients satisfied with telemedicine visits facilitate provider use of TMH		“The results of this project demonstrate that telehealth is a feasible and acceptable strategy for delivering follow-up PMH appointments for patients of a community-based clinic who reside in rural locations. Post intervention surveys revealed patient satisfaction with the utilization of telehealth [8].”	
	Patients interested in doing telemental health care	2		[32, 45]		Mention that TMH is facilitated when patients show interest in its use		“Lal and colleagues (2020) reported that 82% of participants indicated interest in a future clinic appointment via video-conferencing, and 75% believed it would be a good tool for unexpected or emergency situations, or when in-person meetings were impossible. Further, 78% of participants in this study reported obstacles to attending in-person sessions and would thus potentially benefit from a virtual option [45].”	
**Concerns**			40				Mention of TMH’s concerning aspects that should be considered in health care delivery		“These concerns included receiving insufficient training to be effective with telepsychology, ensuring the patient’s safety or addressing the needs of remote patients in crisis, protecting the privacy of patients, considering ethical issues that could result from using telepsychology, and being uncertain about the effectiveness of treatment when not conducted in person [46].”
	Patient and provider barriers	12		[9, 11, 17, 18, 22, 37, 40, 47-51]		Discussion of telemental barriers that relate to both providers and patients such as having to learn to use telemedicine or their preferences		“Practical considerations affecting the uptake of the new COVID-19 MBS-telehealth items include: understanding of the usage of the items; technology, accessibility and cybersecurity; patient and psychiatrist consultation preferences; appropriateness of telehealth for individual patients’ circumstances and suitability to develop empathy and rapport [22].”	
	Patient-centered barriers	9		[17, 22, 25, 33, 38, 41, 50, 52, 53]		Discussion of telemental barriers that relate predominantly to patients such as the inability to attain prescriptions or inadequacy of TMH for certain patients		“However, telehealth may be less useful in patients with significant social disadvantage, and severe mental illnesses that impair cognitive abilities and insight such as schizophrenia and major neurocognitive disorder; further research for this population is needed [24].”	
	Provider-centered barriers	19		[9, 17, 18, 22, 24, 28, 29, 32-34, 41, 44-46, 48, 49, 52-54]		Discussion of telemental barriers that relate predominantly to providers such as loss of in-person contact, not using existing guidelines, and loss of visual cues		“In this situation, the GP may be little motivated to add another tool to his/her professional armamentarium, especially because of time constraints and crowded clinics [54].”	
	Environment and technology	18		[17, 18, 21, 22, 24, 25, 27, 32-34, 38, 41, 42, 50, 52, 53, 55, 56]		Discussion of telemental barriers relating to the technology needed or environment, such as technical aspects and type of device used		“Older adults, individuals from low SES populations with less experience with technology and individuals for whom the technology navigation tools may be in a second or third language, may require considerable emotional reassurance as well as technical assistance as they interact with a virtual doctors’ office. Normalizing the difficulty with new technology is particularly important as we meet patients for the first time who otherwise might feel they are being judged as ‘cognitively incapable’ due to their difficulty with the telehealth technology [17].”	
	Compliance, regulatory, security, and reimbursement	20		[9, 17, 19, 20, 22, 25-28, 31-34, 36, 41, 46, 47, 52, 55, 57]		Mention of how the legal and policy structures impact TMH, especially in terms of cost/payments, security, and privacy		“In this study, the problems related to ethical, legal, accountability, and regulatory implications were cited as challenges by almost all respondents. The major issues stated by patients were privacy and confidentiality, ethical violations, security and hacking, and data ownership [57].”	
	General discussion of barriers	3		[27, 28, 58]		Discussion of nonspecific barriers in how they negatively impact the use of TMH		“Telehealth technology is a tool to support the navigation of barriers currently facing ABA providers; however, substantial challenges (eg, how to structure a credible telehealth session) need to be overcome for telehealth to achieve its full potential within the field of ABA [58].”	
**Changes advised**			76				Discussion of recommendations, advice, and guidelines regarding successful implementation and use of TMH by providers		“Most published PMH APRN telepsychiatry literature predominantly focuses on clinical services across pediatric populations and the rural and urban underserved and was published in the past 15 years. Academic partnerships with primary care, hospitals, shelters, and federal clinics implemented and disseminated successful telepsychiatry consultations and scheduled services, but it is unclear if these programs were sustainable long-term despite short-term feasibility. Other services were usually on a small scale and included scheduled weekly or monthly consultations across primary care clinics [59].”
	Technology-related recommendations	23		[7, 11-13, 16-18, 20, 25, 31, 33, 36-39, 47, 52, 60-65]		Mention of hardware or software needed to successfully use telemedicine		“Consider information governance issues and the information technology (IT) system that you and your patient will be using. Prepare the patient: ensure the patient has relevant information before the consultation. Prepare yourself: be familiar with the IT system you will use [12].”	
	Interacting with patients and developing therapy plan	22		[7, 10-13, 17, 18, 24, 25, 28, 31, 34-37, 40, 42, 43, 55, 63, 65, 66]		Mention of best practices and advice for providers before, during, and after telemental visits with patients		“Know who fits with which modality (consider research, repeated emergencies, tendency toward crises, access to resources, patient's comfort, etc.); refer to in‐person services when necessary; develop plan in case patient's inappropriateness emerges after onset of telepsychology services [7].”	
	Types of guides	24		[7, 10, 12, 16-19, 24-26, 32, 34-38, 42, 49, 55, 58, 63, 67-69]		Any type of guide or checklist that is presented within an article, such as step-by-step, general rules of thumb, and templates		“In this tutorial, we provide practice recommendations, task analyses, and a curated list of Zoom walk-throughs to help behavior analysts construct conceptually systematic learning opportunities in their direct telehealth services. Leveraging teleconferencing features to provide behavior-analytic intervention directly to consumers could spur future research to support these need-inspired practices and guide telehealth applications during and beyond the current pandemic [67].”	
	Further research	59		[8-12, 14, 17-22, 24, 26-28, 31-34, 37-42, 44-47, 50-64, 67, 68, 70-81]		Mentions of the need for further research on a telemedicine topic that would impact the successful implementation or use of TMH		“While telemedicine services in psychiatry have increased significantly, limited quantitative data exist that examine the training and implementation of the service. It is important that the ability of the practitioner, internal and external barriers to use, and patient satisfaction are explored when considering widespread implementation [70].”	
	Patient orientation of telemental health care	23		[7, 10-13, 16-19, 37, 40, 44, 48, 50, 53, 55, 56, 61, 63-66, 69]		Discussion of how providers may prepare and best inform patients in regard to TMH		“Proper releases of information should be completed for the patient support person prior to the start of CPT. Service providers should also have information for other critical community resources, such as hospitals, the police department, and shelters, accessible in case they become clinically necessary. Providers and patients should establish an agreed-upon back-up plan for service delivery in the event of technology failures, such as rescheduling or completing the session via phone to ensure minimal interruptions to treatment [10].”	
	Laws and regulation	28		[7, 10-20, 27, 29, 33, 35, 37, 44, 47, 51-53, 61, 63, 64, 66, 67, 69]		Mention of legal or ethical aspects of TMH, including consent forms and provider knowledge of regulations		“Consent must be obtained within the framework of the legal provisions enforced by each country. It is essential to know the applicable relevant laws and the regionality of these laws. As a general rule, when practices take place across countries and there is a conflict between the laws of the two countries, the laws of the patient’s location prevail over the laws of the neuropsychologist’s location [13].”	
	Telemental education and integration into practice	42		[7, 9-12, 14, 16-20, 24-30, 33, 34, 37, 38, 40, 42, 44-46, 50, 52-56, 58-60, 63-67, 70]		Discussion of the need for education in telemedicine and learning how to integrate telemedicine and innovative techniques into a practice		“The impact of telehealth on trainee education is unknown. However, given that telehealth is likely to continue to be used to deliver care in some capacity, training educators in virtual teaching strategies and teaching learners to deliver virtual care and perform virtual observations of children is important [60].”	
	Consider evidence of access, culture, and disparities	32		[7, 9, 11, 13, 16-18, 21, 22, 27-29, 31-34, 44, 46-48, 51, 55, 60, 61, 63-65, 67-69, 78, 79]		Discussion of the accessibility, socioeconomic and sociocultural aspects, and health disparities and how they should be considered when implementing TMH		“Neuropsychologists and other health care professionals must be mindful of these disparities and share responsibility in advocacy efforts to ensure equitable access to needed resources in marginalized or isolated communities [27].”	

^a^TMH: telemental health care.

### Themes Identified

#### Facilitators

The Facilitators theme (n=36) predominantly comprised subthemes on how telemedicine increased vulnerable patients’ access to care (n=21) [8-10, 14, 16, 17, 19-22, 24-34]; reduced barriers such as travel, costs, and stigma (n=17) [8, 9, 14, 16, 21, 22, 25, 27, 30-38]; and enabled effective and innovative health care, including coordination of care (n=13) [8, 9, 16, 17, 21, 22, 30, 32-35, 37, 39]. For example, Dexter [14] noted in the letter to the editor that telemedicine may increase accessibility for both patients and providers, as some psychiatric mental health nurse practitioners would no longer need to relocate to deliver care in areas lacking such personnel. In another example, Schroeder’s [9] review on TMH adaptations pointed out that providers can see more patients due to this increased accessibility—partially because transportation and other physical barriers are removed for patients. The subtheme regarding telemedicine removing barriers (n=17) identified general costs, child care, time, stigma, and transportation as specific barriers eased by the use of telemedicine [8, 9, 14, 16, 21, 22, 25, 27, 30-38].

A total of 13 articles identified TMH care as bringing a level of convenience to both providers and patients [8, 9, 16, 17, 21, 22, 30, 32-35, 37, 39], 6 noted the loosening of regulations [24, 28, 39, 42-44], another 6 included subthemes of patients being satisfied with their TMH visit [8, 16, 24, 31, 34, 37], and 2 highlighted the importance of patients being interested in engaging with TMH care [32, 45].

#### Concerns

We identified 6 subthemes in Concerns (n=40). The most populated subtheme was Compliance, Regulatory, Security, and Reimbursement (n=20) [9, 17, 19, 20, 22, 25-28, 31-34, 36, 41, 46, 47, 52, 55, 57], which included regulatory and cost structures directly impacting the ability of providers to offer TMH care and patients’ receipt of such care safely. Provider-Centered Barriers (n=19) [9, 17, 18, 22, 24, 28, 29, 32-34, 41, 44-46, 48, 49, 52-54] included loss of visual cues and specific audio issues during visits (n=5) [9, 18, 24, 29, 32], general loss of in-person contact (n=4) [24, 33, 42, 45], and new provider users not using or understanding existing guidelines (n=5) [22, 28, 34, 48, 53].

The Environment and Technology subtheme (n=18) [17, 18, 21, 22, 24, 25, 27, 32-34, 38, 41, 42, 50, 52, 53, 55, 56] included the technical aspects of TMH services related to hardware, software, and users’ technological literacy (n=10) [17, 18, 22, 25, 32-34, 38, 41, 56], as well as the inaccessibility of TMH care for certain patient demographics (n=10) [17, 18, 21, 24, 27, 32, 33, 38, 50, 52].

The subthemes of Patient-Centered (n=9) [17, 22, 25, 33, 38, 41, 50, 52, 53] and Patient-And-Provider (n=12) [9, 11, 17, 18, 22, 37, 40, 47-51] barriers included specific difficulties patients face in TMH care, including the need to align telemedicine preferences with their providers and learn how to use it. For example, Dinakaran et al [25] pointed out the incomplete care a patient may face when receiving telepsychiatry without access to prescriptions for therapy. One topic, found in 7 articles, mentions the Patient-Centered Barriers subtheme, noting that TMH care may not be adequate for certain patients due to their specific needs related to symptoms or therapy types [17, 22, 33, 38, 41, 50, 52].

#### Changes Advised

We identified a total of 8 subthemes within Changes Advised (n=76). The most prominent subtheme was prompting Further Research (n=59) [8-12, 14, 17-22, 24, 26-28, 31-34, 37-42, 44-47, 50-64, 67, 68, 70-81]. Topics within this subtheme included the need for evidence-based outcomes to guide future guidelines and best practices (n=35) [8, 10, 11, 14, 17, 20, 24, 28, 31-34, 44-47, 50, 52-54, 56-59, 61, 62, 64, 67, 70, 71, 73, 75, 76, 78, 80], the need for clinical validation of assessments and tools used in telemedicine (n=17) [11, 17, 18, 31, 32, 34, 44, 51, 52, 55, 62, 63, 67, 75, 76, 79, 80], and the need for testing innovative interventions (n=10) [31, 33, 44, 50, 51, 54, 55, 58, 74, 79].

Another dominant subtheme is Telemental Education and Integration Into Practice (n=42) [7, 9-12, 14, 16-20, 24-30, 33, 34, 37, 38, 40, 42, 44-46, 50, 52-56, 58-60, 63-67, 70]. While formal, continuing, and organizational education was discussed as a topic (n=17) [7, 11, 16-18, 25, 27, 34, 36, 37, 45, 46, 63, 64, 66, 67, 70], the dominant focus of this subtheme was the need for innovative methods to deliver traditional in-person treatments via TMH (n=32) [7, 9-11, 14, 17-19, 24-30, 33, 34, 36-38, 42, 44, 45, 50, 52-56, 58, 66, 67]. Additional topics included the use of new and existing technology alongside telemedicine to support high-quality care (n=10) [10, 11, 18, 19, 33, 42, 44, 54, 66, 67], as well as traditional in-person exercises, which require their own guidelines when applied via telemedicine (n=6) [18, 27, 34, 45, 66, 67].

The subtheme of Changes Advised included the types of guides provided and requested (n=24) [7, 10, 12, 16-19, 24-26, 32, 34-38, 42, 49, 55, 58, 63, 67-69]. The resulting articles covered a variety of topics, such as a detailed protocol for using a specific telemedicine platform (n=1) [67], and step-by-step protocols for specific treatments (n=9) [10, 34, 35, 37, 49, 55, 58, 63, 69], including directed family play and activity-based instruction. Nearly one-third (24/76, 32%) of the resulting articles provided a functional guide, protocol, or detailed checklist.

While some papers did not include guidelines, others provided specific instructions on setting up a TMH care process with patients. Patient Orientation of Telemental Health Care (n=23) [7, 10-13, 16-19, 37, 40, 44, 48, 50, 53, 55, 56, 61, 63-66, 69] was a subtheme that covered topics ranging from ensuring patient safety through backups and emergency plans (n=10) [7, 11, 12, 25, 40, 50, 55, 65, 66, 69] to clearly defining services for patients before a TMH visit (n=10) [10, 18, 37, 40, 48, 53, 55, 63-65].

A similar subtheme of Interacting With Patients and Developing Therapy Plans (n=22) [7, 10-13, 17, 18, 24, 25, 28, 31, 34-37, 40, 42, 43, 55, 63, 65, 66] addressed topics related to both providers and patients being prepared for TMH care visits. The most common topic within this subtheme was determining the suitability of telemedicine (n=14) [7, 10, 11, 13, 17, 18, 24, 25, 31, 35, 37, 40, 63, 66] based on the patient (n=9) [7, 10, 11, 13, 17, 18, 24, 31, 40], as well as on the type of consults or assessments required for the patient’s treatment (n=4) [13, 17, 18, 37]. This subtheme also included providers ensuring they are prepared for a telemedicine session ahead of the visit (n=8) [11-13, 18, 36, 37, 40, 65].

The subtheme of Consider Evidence of Access, Culture, and Disparities (n=22) [7, 9, 11, 13, 16-18, 21, 22, 27-29, 31, 33, 44, 46-48, 63, 68, 78, 79] encompasses sociocultural and socioeconomic considerations regarding the use and implementation of TMH care (n=19) [9, 11, 16, 17, 21, 22, 27-29, 31, 33, 44, 46-48, 63, 68, 78, 79]—including tangible requirements such as equipment (n=7) [11, 32, 33, 60, 61, 63, 78], the necessity of the right space/environment for a TMH visit (n=8) [7, 11, 13, 17, 18, 55, 64, 69], and the availability of interpreters or providers who can use patients’ preferred languages (n=3) [27, 44, 63].

The subtheme of Technology-Related Recommendations (n=23) [7, 11-13, 16-18, 20, 25, 31, 33, 36-39, 47, 52, 60-65] includes topics such as the basic technology requirements, including sufficient physical infrastructure and reliable equipment for telemedicine (n=14) [11-13, 16-18, 31, 33, 36, 37, 60, 61, 64, 65], as well as the need to increase comfort with using technology during mental health care visits (n=9) [7, 11, 18, 37, 47, 61, 63-65]. Within the topic of technology requirements, 3 articles specifically highlight the need for reliable equipment [11, 33, 61], and 5 emphasize the necessity of infrastructure and support [11, 13, 16, 17, 33]. Other topics include successful system integrations and health care technologies (n=4) [17, 20, 62, 64], recommendations on the types of devices patients should use based on the treatment and assessment (n=4) [13, 17, 25, 37], and the importance of telemedicine platforms having accessible user interfaces (eg, language preference options) [17].

Finally, 28 articles [7, 10-20, 27, 29, 33, 35, 37, 44, 47, 51-53, 61, 63, 64, 66, 67, 69] specifically advised on Laws and Regulations regarding TMH care, including ethical and social issues (n=15) [7, 15, 17, 18, 25, 27, 29, 33, 37, 51, 53, 61, 63, 66, 67], patient consent (n=9) [7, 10, 11, 13, 17, 18, 25, 29, 63], providers’ knowledge of laws (n=9) [7, 11, 12, 16, 17, 25, 52, 63, 66], and the use of Health Insurance Portability and Accountability Act (HIPAA)–compliant platforms when delivering telemedicine (n=3) [11, 64, 69]. Another topic within laws and regulations includes reimbursement for telemedicine visits and ensuring that patients are informed about how TMH care may be billed differently (n=5) [11, 17, 20, 27, 47]. Bilder et al [17], Voulgaris et al [35], and Batastini et al [63] emphasized that their recommendations should never supersede existing laws, ethical standards, or organizational guidelines, such as those from the APA. All 28 articles adhered to current regulatory frameworks in the context of their advised changes. Additionally, 5 articles explicitly highlighted the importance of addressing socioeconomic disparities when discussing changes to ethical guidelines and expectations related to telemedicine. These disparities must be considered both in the implementation of TMH care and in the development of future guidelines [17, 27, 29, 33, 63].

## Discussion

### Principal Findings

This scoping review aimed to identify the types of guidelines and recommendations available to providers in peer-reviewed sources, highlight the most common recommendations presented, and examine notable benefits and challenges associated with TMH guidelines. We analyzed TMH guidelines and biomedical recommendations published between 2020 and 2024, identifying key themes based on their frequency across the literature, and categorized the types of guidance available to providers during that time. Through content and thematic analyses, this study identified 3 main themes along with their associated subthemes and topics. Our findings highlight Concerns and Facilitators of TMH that were directly addressed by published guidelines, as well as overarching patterns in TMH guidance from 2020 to 2024. Using a qualitative approach, we presented these elements not only by frequency but also by their relevance within the broader thematic structure. Notably, our review found that the most frequently cited Facilitators of TMH care was its ability to increase access for isolated and vulnerable patient populations [8-10, 14, 16, 17, 19-22, 24-34]. Concerns focused on Provider-Centered Barriers, including the lack of visual and physical cues during sessions, as well as environmental and technological limitations that made TMH care inaccessible for certain patient populations [9, 17, 18, 22, 24, 28, 29, 32-34, 41, 44-46, 48, 49, 52-54]. The Changes Advised theme responded directly to these concerns by presenting solutions and actionable recommendations aimed at addressing the identified challenges. Among the most prominent recommendations identified in the 76 articles were the call for Further Research across all aspects of TMH care and the need for comprehensive telemedicine education for providers at various levels [8-12, 14, 17-22, 24, 26-28, 31-34, 37-42, 44-47, 50-64, 67, 68, 70-81].

### Guidelines, Protocols, and Recommendations Published for Providers During the Early Pandemic

While all included articles offered at least some form of recommendation, only a few provided concrete, step-by-step guidelines. Among these, some offered guidance specific to certain treatments [10, 34, 35, 37, 49, 55, 58, 63, 69], 1 provided instructions for using the Zoom platform for telemedicine delivery [67], and others outlined general best practices for providers [7, 12, 16-18, 24, 25, 32, 36, 37, 42, 55, 63, 67-69]. This range of guidance highlights the extent to which TMH care can differ from traditional in-person visits.

The guidelines generally appeared to draw on information and rules of thumb from trusted organizational resources, serving as a conglomeration of established best practices. Across the reviewed literature, we identified 47 distinct organizations cited as sources for developing TMH recommendations and guidance. Several organizations were referenced multiple times, including the APA, ATA, IOPC, CMS, CDC, the Institute of Medicine, and the Telemedicine and Psychiatry Operational Guidelines 2020 [7-19, 63]. The reliance on multiple trusted organizational resources in place of direct clinical evidence highlights a significant need for more empirical research that links specific telemedicine practices to measurable outcomes. Furthermore, the higher number of original research articles published between 2020 and 2021—compared with the 3 subsequent years combined—suggests that while evidence is beginning to emerge, the momentum for generating new data may have slowed. The guidelines we assessed covered a broad range of topics, from providing patient orientation for telemedicine to outlining best practices for developing therapy plans and engaging with patients during virtual visits. These resources seem to serve as foundational tools—offering insights into essential technology requirements, availability, and general checklists to help ensure the delivery of high-quality care. However, to truly meet the complexities of telemedicine, more detailed and tailored guidelines will be needed. In the future, comprehensive TMH protocols may need to be developed not only for individual countries but also adapted at the state or territorial level, depending on specific regulatory frameworks. These future guidelines must also account for therapist compliance with local laws and professional standards.

In the review, we found 9 step-by-step guides focused on specific TMH treatments. For example, Smith et al [37] developed a guide for virtual family play therapy, focusing on “recommendations for assessment, therapy structure, therapist roles, session preparation, and how to use virtual tools to enhance treatment.” These guides are ultimately recommendations and have yet to be assessed for validity. As more step-by-step guides become available, there should be a parallel initiative to evaluate their quality and validity. Such efforts would help ensure that providers deliver evidence-based, high-quality TMH care. Developing these guidelines would also support the systematic provision of care that incorporates innovative techniques and remains ethically sound.

Finally, the guide on how to use a particular telemedicine platform successfully for TMH care addressed the “gap in support” [67]. While telemedicine platforms may offer their own guides, question-and-answer sections, and other customer support services, it is important to explore platforms specifically designed to facilitate TMH practices. Within these platform-specific guidelines, the development of specialty resources—such as a TMH guide—would help ensure high-quality care and address many of the concerns raised by providers, including technical aspects of telemedicine use and how to recognize visual and auditory cues from patients [37]. After all, platforms may have different features and technical aspects that can impact TMH care. For example, platforms have been shown to exhibit acoustic differences [82]. If providers are unaware of such variations in audio or visual quality, their ability to deliver effective care may be hindered.

### Further Research Crucial for the Future of TMH Care

It is unsurprising that 59 of the 76 (78%) peer-reviewed publications stated the need for Further Research to support the success of TMH care; however, the necessity of acting on this recommendation is critical. While a general call for robust telehealth evaluation has been cited [24, 31, 32, 34, 38, 44, 52, 55, 60, 61, 64, 68, 70, 77, 78, 80], our review highlights the many specific facets of TMH care that still require investigation. Evaluations of TMH care must consider both patient and provider perspectives [61]. Researchers and clinicians should strive to evaluate and validate the assessments, exercises, and treatments used in TMH care to ensure the highest quality of care and to identify best practices [11, 17, 18, 31, 32, 34, 44, 51, 52, 55, 62, 63, 67, 72, 75, 79, 80]. Although original research publications providing evidence-based recommendations are on the rise, the guidelines themselves have not undergone significant changes—though the ATA and APA guidelines have been consistently updated. While major revisions may not be necessary, a maintained and regularly updated resource list of evidence-based telemedicine practices should be available to providers. As Sperling et al [44] noted, “scientific studies examining the reliability and validity of tele-np-t [tele-neuropsychological testing] are outnumbered by published editorials, white papers, descriptive reports of institutional practices, and/or practice surveys related to tele-np...[it is] imperative that professionals...turn their expertise and attention to conducting well-designed experimental studies.” Naturally, evaluations must be conducted via telemedicine and across different countries to assess and account for the impact of language, culture, and socioeconomic factors in TMH care [62]. As Law et al [51] pointed out, different TMH techniques should be evaluated and compared to determine which telemedicine approaches work best for specific patients, providers, and scenarios.

The articles included in the scoping review highlight that, although telemedicine research has been conducted in the past, the existing evidence does not adequately support current practice. With the rapid uptake of telemedicine due to the COVID-19 pandemic, it now encompasses far more dimensions and considerations than the current body of evidence can fully address. As Finley et al [59] and other authors have noted, while the literature demonstrates positive, evidence-based outcomes with TMH care, further large-scale research is needed to determine whether these outcomes are generalizable beyond the original studies—and whether TMH practices are ultimately “sustainable long-term despite short-term feasibility” [32, 33, 52, 59, 78]. Further research is needed on how guidelines, protocols, and best practices are developed and implemented at professional, organizational, and national levels. Pre–COVID-19 studies explored telemedicine as an emerging technology; however, its current state reflects widespread adoption without clear guidance for direct integration into practice.

### Impact of Laws and Regulations on TMH Practice

While COVID-19 served as the catalyst for significant regulatory upheaval and changes in telemedicine-related laws to ensure the protection of both providers and patients, none of the articles proposed changes to the laws and regulations. Of the 28 articles that discussed laws and regulations, all focused on complying with current legal requirements. There was an emphasis on providers’ understanding of the laws in their region [7, 11, 12, 16, 17, 25, 52, 63, 66] and the idea that any recommendations presented in the articles should never supersede laws or ethics [17, 35, 63]. However, no mention was made of the need for regulatory changes. Interestingly, several articles recommended reviewing and revising the ethics surrounding TMH care provision [14, 15, 17, 18, 29, 33, 51, 53]. We infer that, due to the unknowns and the use of emergency protocols by nations, authors chose to omit recommendations for regulatory and policy changes. However, the suggestions for revisions in ethical standards indicate a need to reconsider legal standards as well. For example, Singh and Sagar [53] noted that, although guidelines may be available, it is not necessarily the case that the practitioner has had the opportunity to adequately train or qualify to provide TMH care [53].

Although we did not observe a direct call for changes in regulatory standards, we did note recommendations for updating patient consent forms and ensuring that patients understand the limitations of TMH care [7, 10, 11, 13, 17, 18, 25, 29, 63]. Fox-Fuller et al [18] emphasized that providers must “weigh ethical considerations when determining” whether TMH care is appropriate and should include potential limitations in care within the informed consent. They also pointed out another recommendation supporting changes to APA standards and the ethical principles of TMH assessments [17, 35, 63]. Such recommendations not only suggest potential policy and regulatory shifts but also highlight the need for telemedicine education, as well as consideration of accessibility and inequities in care. Practice guidelines focus on reminding providers to be mindful of laws and regulations but generally do not offer recommendations for legal and regulatory changes, as they are intended to guide care.

### Telemedicine Training for Providers and Innovative Ways to Deliver Treatments

Telemedicine may not be a new technology, but its widespread use became prominent in 2020. It incorporates many levels of innovation, such as the integration of new technologies with telemedicine [10, 11, 18, 19, 33, 42, 44, 54, 66, 67], the development of new exercises for patients [18, 27, 34, 45, 66, 67], and the screening of patients for successful telemedicine use [7, 18, 42]. For a provider to use these innovations effectively, some form of training is necessary. Interestingly, only 17 out of the 76 (22%) articles directly discuss the need for telemedicine-specific training. This result may reflect both the novelty of the widespread use of telemedicine and the emphasis on developing guidelines and recommendations for immediate use by providers.

Some articles addressed the need for formal educational processes, such as medical school education or continuing medical education [7, 11, 16-18, 25, 27, 34, 36, 37, 45, 46, 63, 64, 66, 67, 70], while others highlighted more immediate forms of education available during the pandemic, such as webinars or consulting with colleagues experienced in telemedicine [11, 17, 18, 36, 63]. The guides and best practices that emphasized greater integration of telemedicine into formal education echo the theme that there is a need for evidence-based outcomes and assessments of validity to develop successful education programs. Tailoring education to specialty practice concerns and needs can be achieved through diligent research [34, 46, 63]. Understanding the necessary adaptations of platforms, exercises, and treatments is essential for TMH care, but it is also important to consider the cultural and socioeconomic aspects of both providers and patients [16, 34]. While knowledge of cultural mannerisms may be critical in care—especially as providers can broaden their patient base through telemedicine—there must also be consideration of patient accessibility and health disparities in the context of telemedicine [16].

### Telemedicine Access, Care Inequities, and Health Disparities

Telemedicine is often credited with increasing access to care for patients and reducing health disparities and inequities [83]. However, with the rise in telemedicine research during the pandemic, inequities and disparities in health became more apparent [84-86]. In our literature search, we found 32 articles that highlighted the need to consider accessibility in various forms—such as the need for interpreters, providers who can communicate in multiple languages, patients requiring the appropriate equipment for successful care, and the need for a distraction-free space [7, 11, 13, 17, 18, 27, 60, 61]. Of these 32 articles, 19 focused on sociocultural considerations. For example, Comer [28] noted that providers need expanded sociocultural training, as “telehealth offers new opportunities to treat individuals in underserved communities.” When providers are unable to adequately meet their patients’ needs, a barrier is created—one that is often most acutely felt by underserved populations [48]. Zhu et al [47] described a revealing need centered on increasing accessibility: “A report published by the Rural Policy Research Institute outlined a need for research to understand the acceptability for long-term TMH service use among rural adults, especially those who are older or racially/ethnically diverse. This also includes studying how to adapt evidence-based treatments to a cultural context and video-based platform.” Once again, the need for more research to determine best practices—especially when considering care inequities and health disparities in telemedicine—is critical. Looi et al [21] also highlighted this vital consideration: “Sensitivity to cultural, health and socioeconomic disparities is also needed to avoid inequities in access.” Not only is sociocultural and socioeconomic consideration necessary in telemedicine implementation, but it should also be integrated into all aspects of the telemedicine visit. This includes providers’ understanding of whether a certain treatment or assessment may be less effective due to cultural factors or obstacles [11]. As telemedicine has become more widely used, new research at larger scales will provide a better understanding of how to effectively increase access to care without exacerbating care inequities and health disparities. With only 19 out of 76 (25%) articles discussing sociocultural and socioeconomic factors in telemedicine implementation, there is a clear need for further research to address this gap in the literature.

### Limitations

This scoping review aimed to provide a snapshot of the guidelines and recommendations produced for providers between 2020 and 2024. Future research could determine whether these themes were addressed through a larger, comprehensive systematic literature review. We intended was to identify what providers could easily find and access within peer-reviewed biomedical databases. As a result, our review is limited to guidelines published between 2020 and 2024. Because of the large number of results from our general search strategy, we conducted a title-and-abstract screening. Outside of the exclusion and inclusion criteria, we did not validate the resulting articles. Future research, particularly systematic reviews, should consider the types of guidelines available for providers; the types of assessments, guidelines, checklists, and techniques that are validated for TMH care; changes in the guidelines over time, especially beyond our restricted 2-year review; and evidence of the sustainable and successful implementation of TMH care in practice.

### Conclusions

This scoping review of 76 articles characterizes the TMH guidelines published between 2020 and 2024 and identifies key, shared recommendations to support the implementation and success of TMH care. We applied systematic qualitative analyses to better understand the most frequently noted themes in the resulting peer-reviewed literature and how they contribute to successful TMH care. This methodology also helped identify how key themes are interconnected and build upon each other. Research at larger scales is essential to develop effective guidelines and protocols for successful therapy plans and to ensure that telemedicine does not exacerbate health inequities. Future research should examine how TMH guidelines have adapted and changed in response to provider and patient needs, how these guidelines address accessibility and inequities, and how they account for regional and international variations.

## Appendix

Multimedia Appendix 1 The PRISMA-ScR (Preferred Reporting Items for Systematic Reviews and Meta-Analyses-Extension for Scoping Reviews) checklist.

## Abbreviations

APA: American Psychological Association
ATA: American Telemedicine Association
CDC: Centers for Disease Control and Prevention
CMS: Centers for Medicare & Medicaid Services
HIPAA: Health Insurance Portability and Accountability Act
IOPC: International Organization of Psychophysiology and Cognitive Sciences
SAMHSA: Substance Abuse and Mental Health Services Administration
TMH: telemental health care
